# Kinetic analysis of HER2-binding ABY-025 Affibody molecule using dynamic PET in patients with metastatic breast cancer

**DOI:** 10.1186/s13550-020-0603-9

**Published:** 2020-03-23

**Authors:** Ali Alhuseinalkhudhur, Mark Lubberink, Henrik Lindman, Vladimir Tolmachev, Fredrik Y. Frejd, Joachim Feldwisch, Irina Velikyan, Jens Sörensen

**Affiliations:** 1 Nuclear Medicine and PET, Department of Surgical Sciences, Uppsala University, Uppsala, Sweden; 2 Department of Immunology, Genetics and Pathology, Uppsala University, Uppsala, Sweden; 3 Research Centrum for Oncotheranostics, Research School of Chemistry and Applied Biomedical Sciences, Research Tomsk Polytechnic University, Tomsk, Russia; 4 Affibody AB, Solna, Sweden

**Keywords:** HER2 receptor, Metastatic breast cancer, Affibody, Dynamic PET, Kinetic modelling

## Abstract

**Background:**

High expression of human epidermal growth factor receptor type 2 (HER2) represents an aggressive subtype of breast cancer. Anti-HER2 treatment requires a theragnostic approach wherein sufficiently high receptor expression in biopsy material is mandatory. Heterogeneity and discordance of HER2 expression between primary tumour and metastases, as well as within a lesion, present a complication for the treatment and require multiple biopsies. Molecular imaging using the HER2-targeting Affibody peptide ABY-025 radiolabelled with ^68^Ga-gallium for PET/CT is currently under investigation as a non-invasive tool for whole-body evaluation of metastatic HER2 expression. Initial studies demonstrated a high correlation between ^68^Ga-ABY-025 standardized uptake values (SUVs) and histopathology. However, detecting small liver lesions might be compromised by high background uptake. This study aimed to explore the applicability of kinetic modelling and parametric image analysis for absolute quantification of ^68^Ga-ABY-025 uptake and HER2-receptor expression and how that relates to static SUVs.

**Methods:**

Dynamic ^68^Ga-ABY-025 PET of the upper abdomen was performed 0-45 min post-injection in 16 patients with metastatic breast cancer. Five patients underwent two examinations to test reproducibility. Parametric images of tracer delivery (*K*_1_) and irreversible binding (*K*_*i*_) were created with an irreversible two-tissue compartment model and Patlak graphical analysis using an image-derived input function from the descending aorta. A volume of interest (VOI)-based analysis was performed to validate parametric images. SUVs were calculated from 2 h and 4 h post-injection static whole-body images and compared to *K*_*i*_.

**Results:**

Characterization of HER2 expression in smaller liver metastases was improved using parametric images. *K*_*i*_ values from parametric images agreed very well with VOI-based gold standard (*R*^2^ > 0.99, *p* < 0.001). SUVs of metastases at 2 h and 4 h post-injection were highly correlated with *K*_*i*_ values from both the two-tissue compartment model and Patlak method (*R*^2^ = 0.87 and 0.95, both *p* < 0.001). ^68^Ga-ABY-025 PET yielded high test-retest reliability (relative repeatability coefficient for Patlak 30% and for the two-tissue compartment model 47%).

**Conclusion:**

^68^Ga-ABY-025 binding in HER2-positive metastases was well characterized by irreversible two-tissue compartment model wherein *K*_*i*_ highly correlated with SUVs at 2 and 4 h. Dynamic scanning with parametric image formation can be used to evaluate metastatic HER2 expression accurately.

## Introduction

The introduction of trastuzumab, a HER2-targeting monoclonal antibody, improved the outcome of HER2-enriched breast cancer treatment dramatically [1, 2]. Still, breast cancer is a heterogeneous disease and HER2 expression may vary considerably not only within a given lesion but also between the primary tumour and metastases [3–5]. Obtaining multiple biopsies in a patient is not always possible, and the need has emerged for a robust imaging technique to estimate HER2 status in cancer patients that is non-invasive, quantitative, and cost-effective and allows for repeated examinations.

One of the most successful classes investigated as HER2-imaging agents is Affibody molecules [6]. The second-generation anti-HER2 Affibody molecules (code-named ABY-025) have high stability, diminished interaction with immunoglobulins, and picomolar affinity for HER2 as well as rapid pharmacokinetics with fast blood clearance, resulting in high tumour-to-background ratios shortly after injection [7–10]. In a pilot study, ^68^Ga-ABY-025 PET uptake quantification suggested a preliminary SUV threshold of 6 for the discrimination of HER2-positive from HER2-negative lesions [11, 12]. SUV is a highly repeatable measurement; however, it relies heavily on the acquisition and reconstruction protocols used [13].

SUV correlated strongly with IHC scores performed on biopsies. However, the liver had the second highest SUV values among normal tissue after the kidneys at 60-240 min post-injection with about 9% of the injected activity retained by the liver [12, 14]. The relatively high liver background uptake might mask the signal from smaller liver metastases, especially those with low to moderate HER2-receptor expression, and that might compromise the accuracy of HER2 scoring by PET. The solution for the improved image analysis in the liver is crucial given that HER2-positive breast cancer subtypes have a higher probability of liver metastasis which, in these subtypes, is the second most common metastatic site, compared to HER2-negative subtypes [15].

SUV does not distinguish between specific and non-specific uptake in receptor-binding radiotracers such as, in this case, ^68^Ga-ABY-025 [16, 17]. A more accurate investigation of the underlying uptake properties requires kinetic modelling. The rate constants obtained from fitting the time-activity curves (TACs) using kinetic modelling can potentially be used to increase contrast by eliminating non-specific background activity found in surrounding tissue. This, in principle, could enhance tumour visualization in these organs [18, 19]. Individual rate constants, or micro-parameters, describing the rate of transport of the tracer between plasma and tissue and between different states (e.g. free and internalized) in tissue can be estimated using compartment models. The gold standard for tracer kinetic analysis is the estimation of these rate constants by non-linear regression of the operational equations describing these compartment models. In case of a tracer with irreversible kinetics, these micro-parameters can then be used to estimate the net influx rate of the tracer, which, in the present case, is assumed to be related to the HER2. Compartment models can be simplified by linearization using basis functions, which allows for a fast calculation of rate constants for each voxel, resulting in parametric images showing the parameter of interest at the voxel level. A simplified linearized method is the Patlak method, which is a graphical analysis technique that allows for the estimation of the net influx rate without giving information on the individual rate constants.

The present study aimed to (1) explore the applicability of kinetic modelling and (2) parametric image analysis for absolute quantification of ^68^Ga-ABY-025 uptake and HER2-receptor expression and (3) how that relates to static SUVs.

## Materials and methods

### Patients

This was a reanalysis of 16 patients with metastatic breast cancer, described in detail elsewhere [12]. Twelve had HER2-positive tumours and 4 had HER2-negative tumours, according to IHC and FISH. Nine patients of the HER2-positive group and all HER2-negative patients had liver metastases. Patient characteristics are presented in Table 1.

**Table 1:** Patient characteristics

	HER2-positive (*n* = 12), number of patients (%)	HER2-negative (*n* = 4), number of patients (%)
Median age	61	66
ER-positive	9 (75%)	3 (75%)
Site of disease		
Locoregional	4 (33%)	2 (50%)
Liver	9 (75%)	4 (100%)
Bone	8 (67%)	3 (75%)
Lung	2 (17%)	1 (25%)
Lymph nodes	3 (25%)	1 (25%)
CNS	3 (25%)	2 (50%)
Contralateral breast	0	1 (25%)
Other	4 (33%)	1 (25%)
Ongoing treatment with trastuzumab	11 (92%)	0
Biopsy post-PET	9 (75%)	3 (75%)

Reprinted from [12]

### Radiochemistry

The GMP compliant manual production of low and high peptide dose ^68^Ga-ABY-025 was published earlier [20]. Briefly, five vials each containing 100 μg of GMP quality ABY-025 (Affibody AB) were used for the labelling synthesis that resulted in the high peptide dose (427 ± 19 μg) radiopharmaceutical. The ^68^Ge/^68^Ga generator (1850 MBq IGG100, Eckert & Ziegler) was thoroughly validated and qualified. Production and quality control were accomplished within 1 h with a high success rate (100%) and radiochemical purity (99.1 ± 0.4%).

### PET protocol

All 16 patients underwent 1 fluorodeoxyglucose ^18^F-FDG PET (GE Discovery ST, GE Healthcare) examination within 14 days prior to the ^68^Ga-ABY-025 PET. Patients were divided into 2 groups. The first group included 10 patients who underwent 1 ^68^Ga-ABY-025 PET examination, while the second group included 6 patients who underwent 2 examinations 1 week apart for a test-retest study, and 1 patient had no detectable lesions; thus, no retest study was performed [12].

Each PET study for patients in both groups began with a 45-min dynamic scan (6 × 10, 3 × 20, 3 × 60, 5 × 180, 5 × 300 s) starting simultaneously with the ^68^Ga-ABY-025 intravenous injection (241 ± 49 MBq). Dynamic scans for all patients were performed over the abdomen, except for 2 patients who were scanned over the chest area considering that they had metastases within that area. Static whole-body scans from the skull to mid-thigh were performed at 2 h and 4 h post-injection for the first group (4 and 5 min per bed position, respectively), while the second group underwent only 1 static scan at 2 h post-injection. Images were reconstructed using settings supplied by the vendor (OSEM with 2 iterations and 21 subsets; corrections for dead time, decay, and attenuation) into a 128 × 128 matrix with voxel size 3.9 × 3.9 × 3.27 mm. A post-processing Gaussian filter of 6 mm was applied, resulting in an image resolution of 7-8 mm.

### Volumes of interest

Volumes of interest (VOIs) were defined manually on the dynamic PET images using the Carimas 2.9 software (Turku PET Centre, Turku, FI). Concurrent CT and FDG PET/CT scans were used to guide VOI definition. The defined VOIs included metastatic lesions in the liver and bone, lung, and lymph nodes, where available. VOIs were also defined over representative parts of healthy organs including the liver, bone marrow, spleen, kidney cortex, gastric ventricle, small intestines, myocardium, and lungs. The aorta was segmented in the frame where the first pass of the radioactivity peaked. The same VOIs were defined manually on the second dynamic PET for the second group of patients and on the 2 h and 4 h post-injection static images. Lesion margins were avoided where possible while defining VOIs to minimize both spill-in and spill-out partial volume effects. One patient was excluded from further analysis due to severe motion artefacts.

The total number of lesions defined in the first group of patients was 24, of which 12 were over 1 cm^3^ and 5 over 2 cm^3^. The total number of lesions defined for the second group was 16, of which 8 were over 1 cm^3^ and 5 over 2 cm^3^.

### SUV

SUVs from decay-corrected static PET study activity concentrations at 2 h and 4 h post-injection in all patients were calculated using Eq. (1):

1 $$ \mathrm{SUV}=\frac{\mathrm{Activity}\ \mathrm{conc}.\left(\frac{\mathrm{Bq}}{\mathrm{ml}}\right)\times \mathrm{bodyweight}\left(\mathrm{g}\right)}{\mathrm{Injected}\ \mathrm{activity}\left(\mathrm{Bq}\right)} $$

SUV mean values were used for comparison with parametric images values.

### Kinetic analysis

**Fig. 1:**
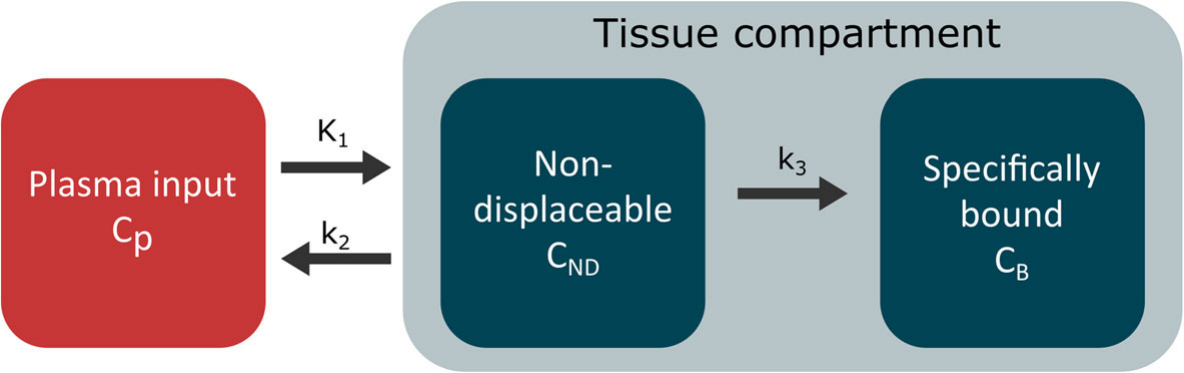
Two-tissue compartment kinetic model used in the analysis of dynamic PET studies with ^68^Ga-ABY-025 in patients with metastatic breast cancer. *K*_1_, *k*_2_, and *k*_3_ represent the transfer rate constants

**Fig. 2:**
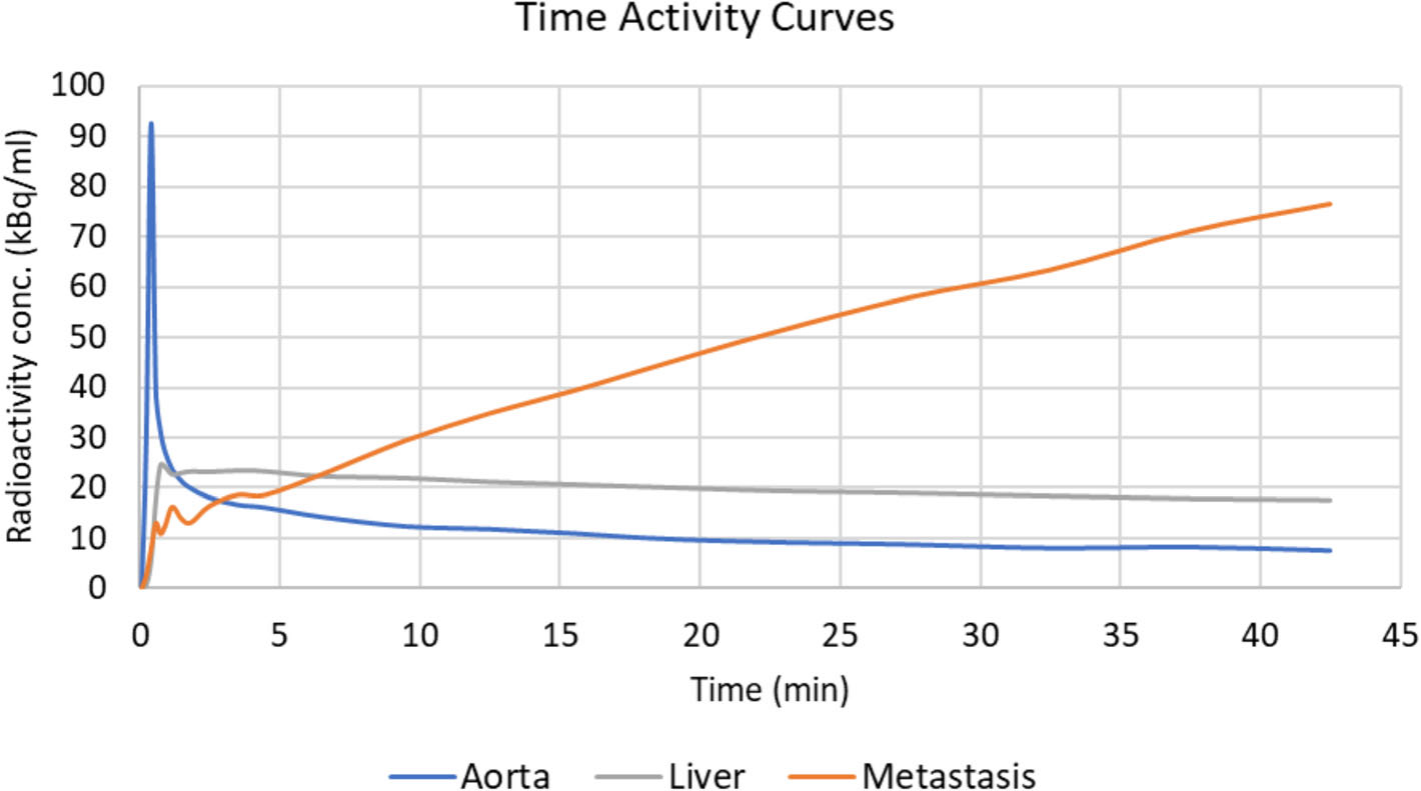
Time-activity curves obtained from dynamic ABY-025 PET of the aorta, liver, and metastasis in a patient with metastatic breast cancer

A VOI-based analysis was performed using a MATLAB program written in-house. Data were analysed using non-linear regression of the operational equations of the single-tissue compartment model (1TC), the irreversible two-tissue compartment model (2TC-3k) (Fig. 1), and the reversible two-tissue compartment model (2TC-4k), all including blood volume as a fitted parameter, as well as the Patlak graphical analysis. The preferred compartment model was established using the Akaike information criterion [21]. These models were used to calculate the rate constants including *K*_1_, which is the delivery constant, and, for the 2TC models, *K*_*i*_, which represent the uptake or influx rate constant. Aorta TACs were used as an input function (Fig. 2). The net influx rate constant was calculated with Eq. (2):

2 $$ {K}_i=\frac{K_1\times {k}_3}{k_2+{k}_3} $$

The *K*_*i*_ cut-off value for HER2 positivity corresponding to the previously defined cut-off SUV value of 6 [12] was determined, along with its confidence interval.

### Parametric images

Parametric images were created using programs written in-house, with aortic TACs as input function. Parametric images of *K*_1_, *K*_*i*_, *V*_*b*_, and *V*_ND_ = *K*_1_/*k*_2_ (delivery, net influx rate, partial blood volume, and non-displaceable volume of distribution) were computed using a basis function implementation of the irreversible 2TC model [19]. In addition, *K*_*i*_ and *V*_*e*_ (distribution volume) images were computed using the Patlak method. The VOIs previously defined on the dynamic images were transferred to the parametric images to obtain the mean values of the kinetic parameters with each VOI.

### Image contrast

For both SUV and parametric *K*_*i*_ images, image contrast was evaluated as the ratio between values in the liver metastases and healthy liver tissue (T/N ratios).

### Statistical analysis

The Mann-Whitney test was used to compare metastatic lesions with normal tissue unless otherwise specified. Wilcoxon’s signed-rank *t* test was used to compare the same parameters between the test and retest studies in the second group of patients. The Bland-Altman plot was created, and intraclass relative repeatability coefficient was calculated for the test-retest group. A *p* value of less than 0.05 was considered statistically significant. Cut-off values were calculated using the best-fit linear regression equation between parametric image values and SUVs, and 95% confidence intervals were calculated using the standard error values of the slope and intercept. All statistical analyses were performed using Prism 7 (GraphPad Software, Inc).

## Results

**Fig. 3:**
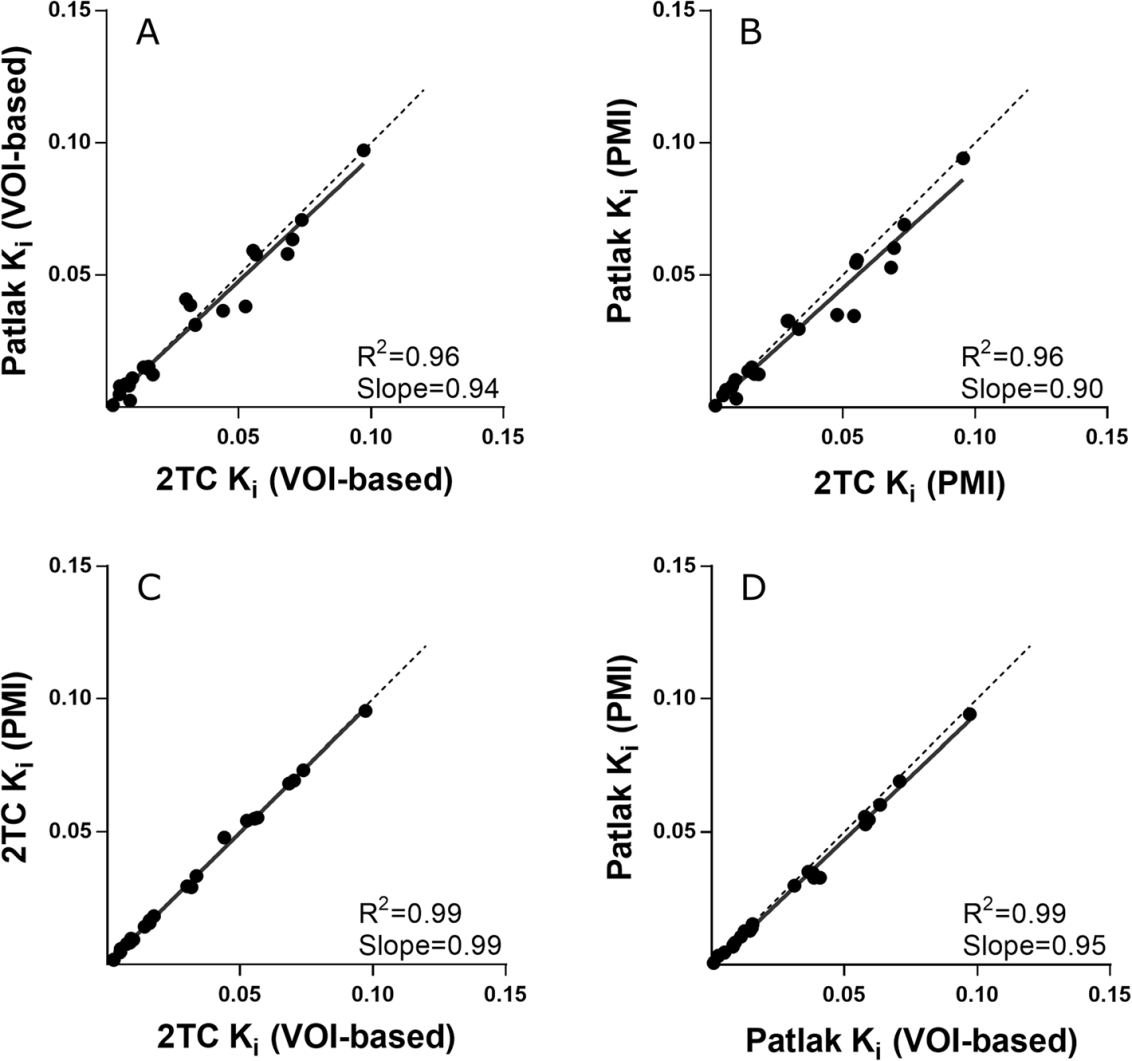
Correlations between **a** VOI-based 2TC *K*_*i*_ and Patlak *K*_*i*_, **b** parametric 2TC *K*_*i*_ and Patlak *K*_*i*_, **c** VOI-based and parametric 2TC *K*_*i*_ values, and **d** VOI-based and parametric Patlak *K*_*i*_ values (PMI, parametric images)

The kinetics of ^68^Ga-ABY-025 were best described by the irreversible two-tissue compartment model (2TC-3k model), giving the lowest Akaike information criterion in 22 out of 40 TACs (55%). Patlak *K*_*i*_ values showed an excellent agreement with 2TC *K*_*i*_ values both for VOI-based analysis and in parametric images (Fig. 3a, b). Both Patlak and 2TC *K*_*i*_ values derived from parametric images agreed very well to their counterparts obtained from the VOI-based analysis (*n* = 24, *R*^2^ > 0.99, and *p* < 0.001 for both correlations; Fig. 3c, d).

**Fig. 4:**
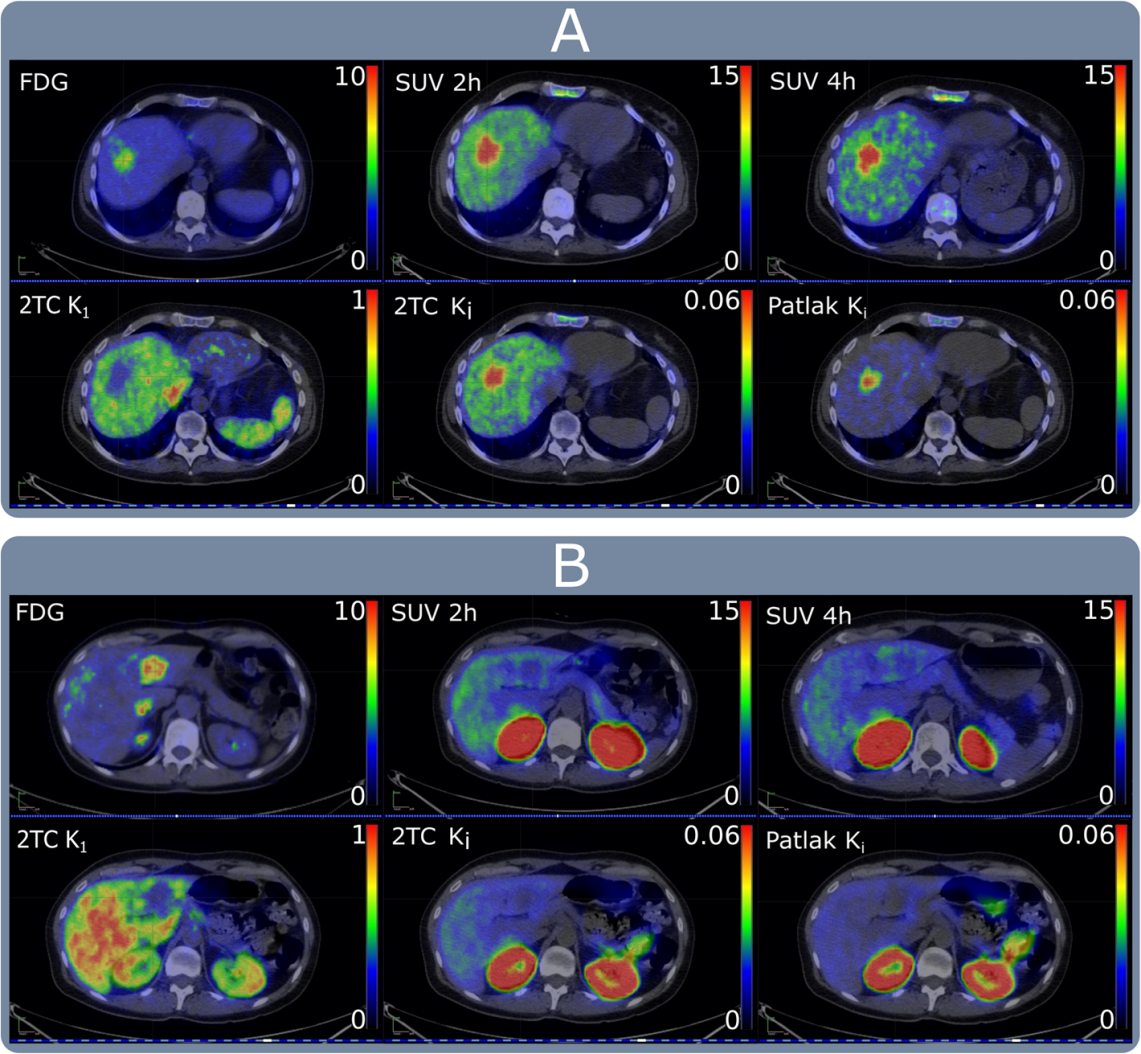
Positron emission tomography (PET) images. **a** In a patient with HER2-positive expression. **b** In a patient with HER2-negative expression. ^18^F-FDG PET images to the left followed by SUV static image taken at 2 h time point after ^68^Ga-ABY-025 injection. 2TC *K*_*i*_, Patlak *K*_*i*_, and (2TC) *K*_1_ are parametric images created from the ^68^Ga-ABY-025 dynamic PET study (*K*_1_, transfer rate constant from plasma to the tissue compartment; *K*_*i*_, influx rate of the tracer into irreversible compartment)

*K*_*i*_ presented good contrast and low background uptake in the normal liver (Fig. 4a). *K*_*i*_ values were 3.7- and 7.1-fold higher in the metastatic lesions compared to the normal liver (Tmax/Nmean ratio 2TC, 3.7 ± 2.8; Patlak, 7.1 ± 7.8). The Patlak Tmax/Nmean ratio was significantly higher (*p* < 0.05) than the corresponding SUV-based ratio (4.2 ± 3.4 at 2 h post-injection). All metastases invariably had lower tracer delivery rates than normal liver and were visualized as cold spots in *K*_1_ images (Fig. 4a, b). Parametric image-based rate constant values and SUV for both liver metastases and normal liver are presented in Table 2.

**Table 2:** ^68^Ga-ABY-025 SUVs and parametric image values in normal liver and liver metastasis, median (range)

	SUV 2 h	SUV 4 h	2TC-3k *K*_1_	2TC-3k *K*_*i*_	Patlak *K*_*i*_
Normal liver	4.9 (3–6.7)	4.8 (3.8–6.1)	0.37 (0.20–0.56)	0.015 (0.006–0.03)	0.008 (0.004–0.016)
All liver met	10 (3.1–32.4)	10 (3.6–39)	0.20 (0.02–0.38)	0.025 (0.009–0.095)	0.025 (0.003–0.094)
*p* value*	< 0.0001	0.008	0.0001	0.019	0.0002

*SUV* standardized uptake value, *2TC-3k irreversible* two-tissue compartment model, *K*_*i*_ transfer rate constant

*Mann-Whitney-Wilcoxon test

^68^Ga-ABY-025 binding in HER2-negative lesions was equal to or lower than the uptake in the normal liver in both *K*_*i*_ and static SUV images (Fig. 4b). *K*_*i*_ values of 0.015 mL/cm^3^/min for 2TC-3k model and 0.013 mL/cm^3^/min for the Patlak model corresponded to the proposed SUV cut-off of 6.0 at 2 h post-injection [12], yielding 95% confidence intervals of 4.4-7.6 (2TC) and 4.8-7.2 (Patlak).

**Fig. 5:**
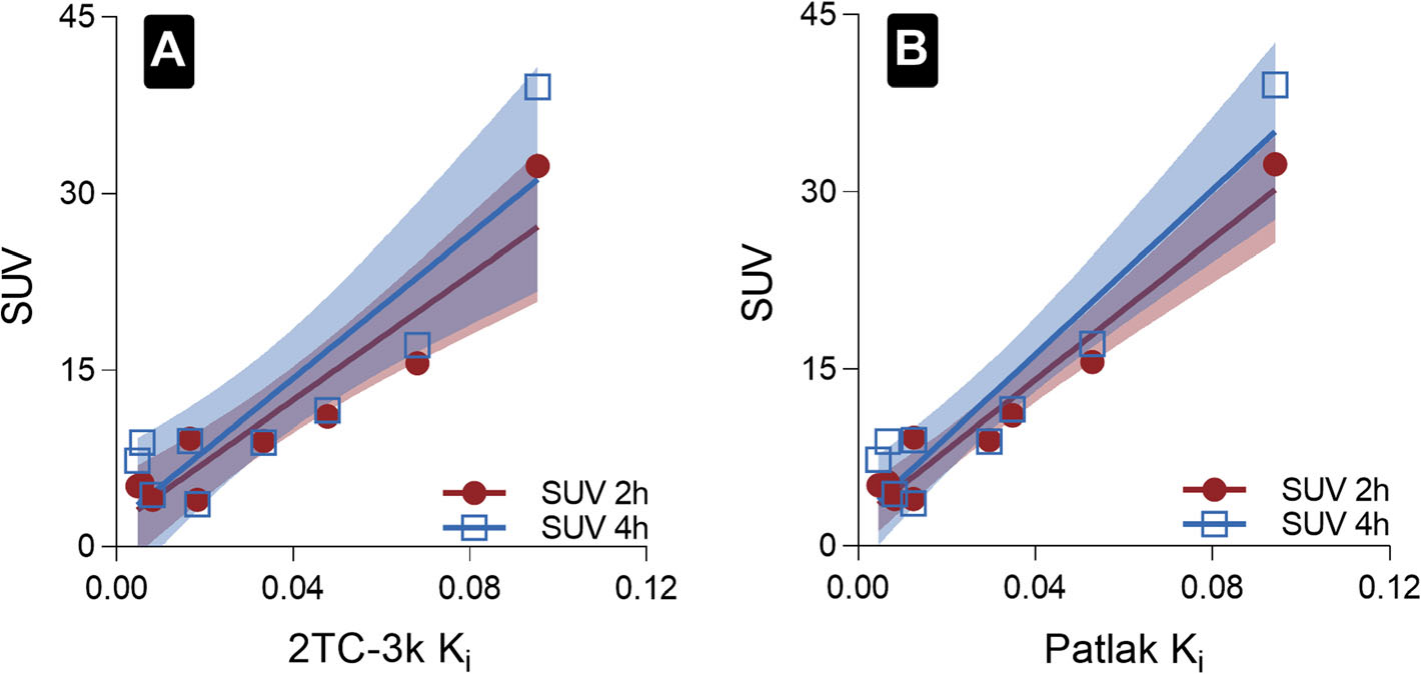
**a** 2TC-3k *K*_*i*_ correlation with SUV 2 h and SUV 4 h (*R*^2^ = 0.87, 0.80 respectively, both *p* < 0.0001). **b** Patlak *K*_*i*_ correlation with SUV 2 h and SUV 4 h (*R*^2^ = 0.95, 0.90 respectively, both *p* < 0.0001). Shaded areas represent 95% confidence band

**Fig. 6:**
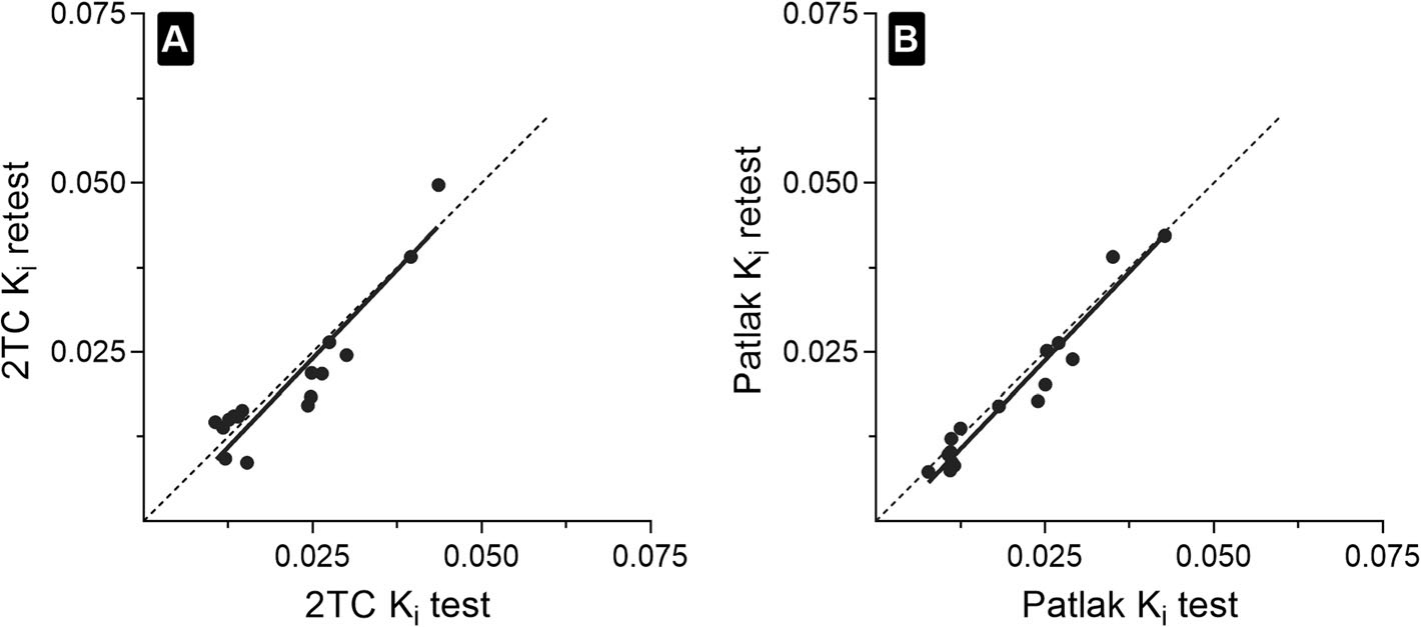
Reproducibility of parametric images shown as a correlation of **a** test-retest 2TC *K*_*i*_ values (*R*^2^ = 0.85, *p* < 0.0001) and **b** test-retest Patlak *K*_*i*_ values (*R*^2^ = 0.94, *p* < 0.0001) for the metastatic lesions in a group of patients rescanned after 1 week (*n* = 5). The solid line is the Deming line, and the dotted line is the identity line

**Fig. 7:**
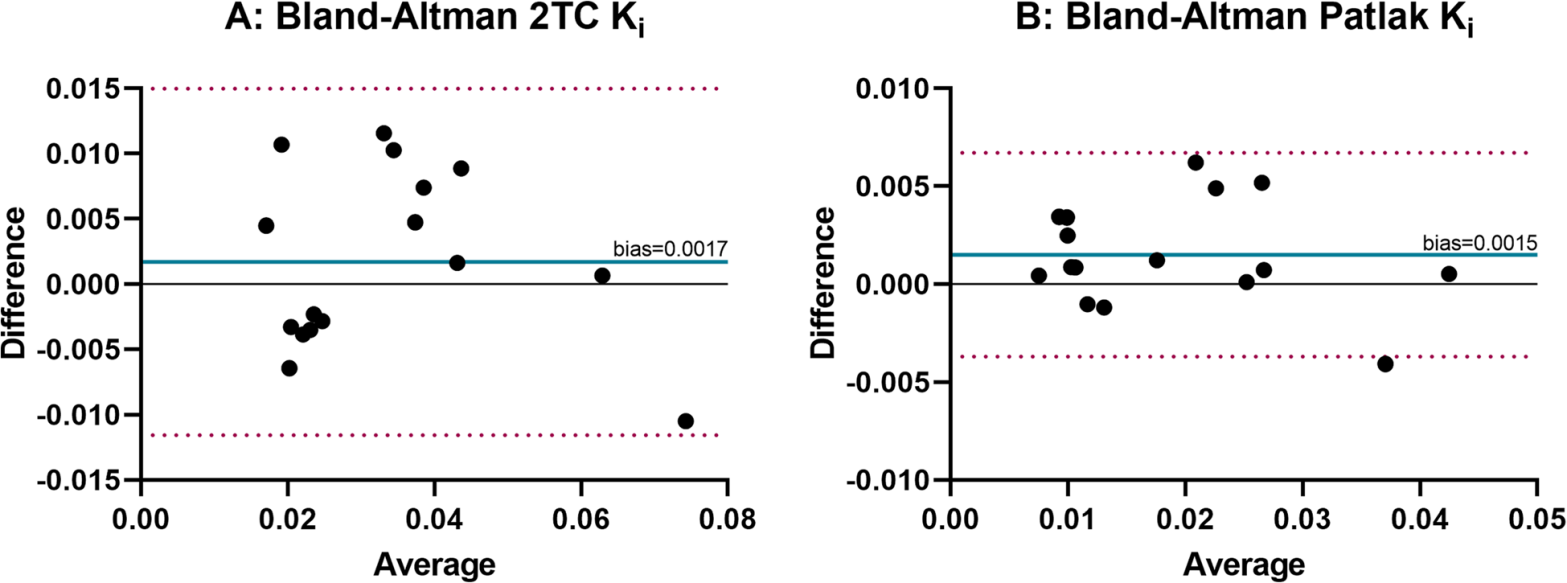
The Bland-Altman plot **a** of two-tissue compartment influx rate (2TC-3k *K*_*i*_) and **b** Patlak *K*_*i*_

Static SUVs obtained at 2 h and 4 h post-injection had a good correlation with parametric image-based *K*_*i*_ (Patlak *R*^2^ = 0.95; 2TC *R*^2^ = 0.87, *n* = 9, Fig. 5). T/N ratios were 4.2 ± 3.4 and 4.7 ± 4.6 for 2 h and 4 h post-injection, respectively (*n* = 10). ^68^Ga-ABY-025 PET test-retest reliability was high as shown by the parametric image-based *K*_*i*_ values in the test-retest group of patients (Pearson *r* ≥ 0.92, *n* = 5) with a relative repeatability coefficient of 30% for Patlak *K*_*i*_ and 47% for 2TC *K*_*i*_ compared to 32% for SUV at 2 h (Fig. 6). Neither systematic nor proportional bias was observed in the test-retest group (Fig. 7).

## Discussion

In this study, we assessed the kinetics of ^68^Ga-ABY-025 with the hypotheses that visualization and determination of HER2 status in liver metastases could be facilitated by eliminating background uptake through creating parametric images of the tracer uptake kinetics, and that simple static SUVs in tumours are acceptable surrogates for the true binding kinetics of the tracer.

The 2TC-3k irreversible model best fitted the data according to the Akaike information criterion. This coincided with the preclinical findings showing almost irreversible kinetic properties of Affibody molecules in HER2-expressing tumours [22]. Since the majority of the measurements in patients were taken from liver lesions, the heterogeneity induced by spill-in from gradually lower uptake in normal liver probably explains why there was a small reversible component in a subset of the measurements.

Both VOI-based kinetic modelling and parametric image analysis returned virtually identical *K*_*i*_ values (*R*^2^ = 0.99, *n* = 24), indicating that VOI-based kinetics are reproducible on the voxel level. The basis function implementation of the 2TC-3k model also provided *K*_1_ images that reflected the delivery rate of the tracer to various tissues, including tumours. This is important when estimating the fraction of the tracer available to cancer lesions for the subsequent computation of the tracer net influx rate *K*_*i*_.

There was a strong correlation between tumour static SUV values and *K*_*i*_ values (Patlak *R*^2^ = 0.95; 2TC *R*^2^ = 0.87, *n* = 9, Fig. 5), indicating that the static SUV images provided a close representation to HER2-specific binding since parametric *K*_*i*_ images reflect the specific uptake solely and eliminate any non-specific binding that increases background uptake. The relatively narrow 95% confidence intervals, especially with Patlak *K*_*i*_, around the proposed SUV cut-off of 6 uphold its use in the upcoming clinical trials and provide a reference to the degree of uncertainty paired with it. Moreover, there was a very good correlation in the test-retest group (Fig. 6), with a relative repeatability coefficient of 30% for Patlak model, indicating high reproducibility of kinetic analysis and similar to the results with static SUV values demonstrated previously [12]. These findings further support the claim that SUV values can be used for the quantification of HER2 expression, as concluded in our previous paper [12]. This will be used in a large multicentric phase II/III clinical trial for non-invasive quantification of HER2 expression in advanced breast cancer using ^68^Ga-ABY-025 PET (NCT03655353) that is currently ongoing.

**Fig. 8:**
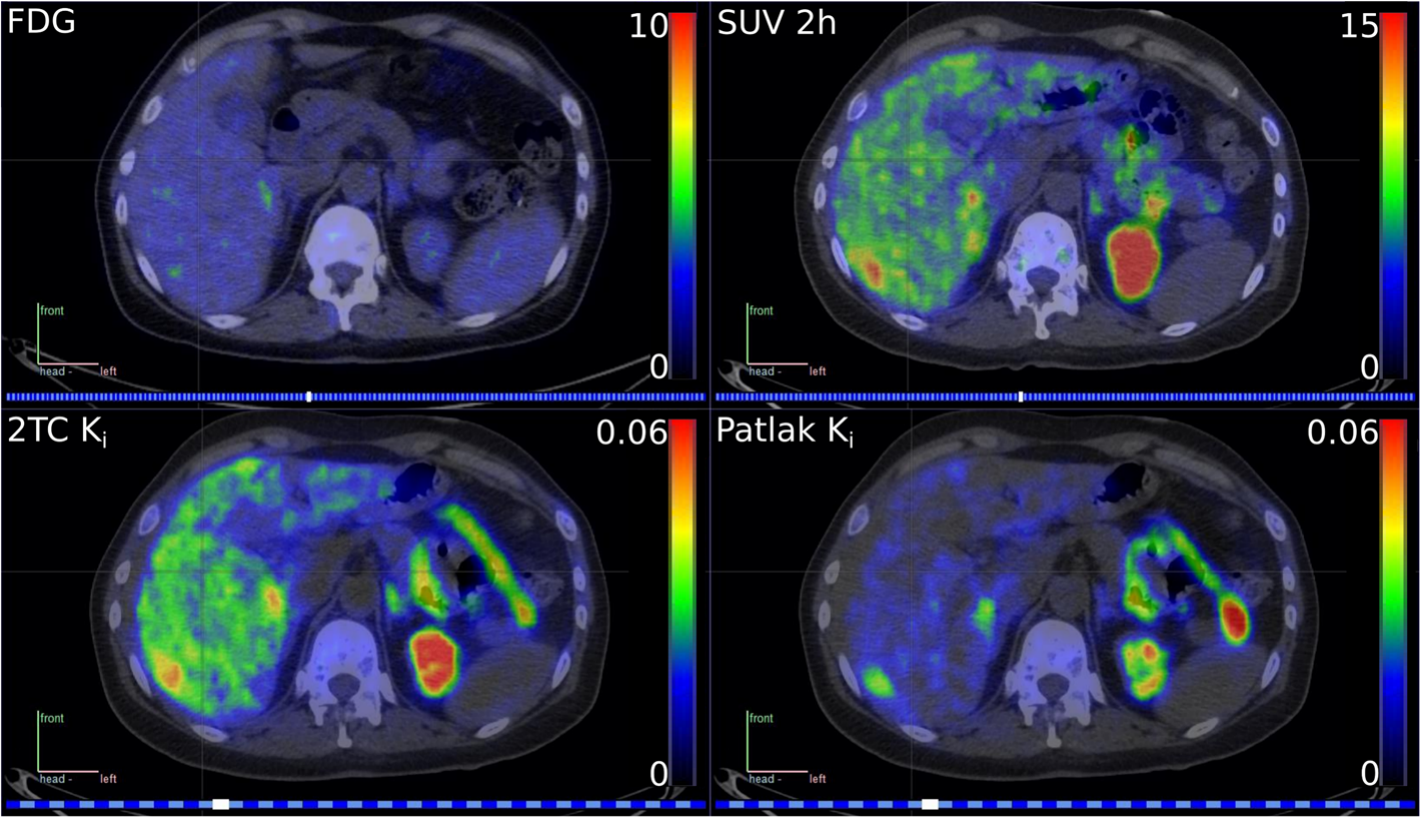
FDG PET and ABY-025 PET SUV 2 h and parametric images of both Patlak *K*_*i*_ and 2TC-3k *K*_*i*_ in a breast cancer patient with multiple small liver metastases

A noticeable degree of specific uptake was recognized in normal liver, further supporting the claim that HER2 receptors are expressed in normal liver tissue [23], although at levels significantly lower than in HER2-positive tumours. The Human Protein Atlas describes liver HER2 expression as moderate, compared to other normal tissues [24]. Liver retention in static PET images is much higher than what can be accounted for by specific binding. This was explained by the very high first-pass extraction with *K*_1_ values significantly higher than for all tumours, probably related to the specialized non-continuous endothelium of liver capillaries. While visualization of the HER2-expressing lesions was possible in both parametric *K*_*i*_ and static SUV images (Fig. 4a), with the feasibility to localize small liver metastases, tumour-to-liver ratios were significantly higher in parametric Patlak *K*_*i*_ images. Thus, parametric imaging might become beneficial in a few clinical cases where it might be difficult to grade the HER2 expression in small metastases due to the high liver background uptake. Because dynamic imaging introduces an additional 45-min scan, the presence of small suspicious liver lesions (< 1 cm) should be known from other imaging modalities beforehand and the resulting HER2 score should be of direct relevance for therapeutic decisions. In terms of contrast, the Patlak method performed better than the 2TC-3k method, mainly because *K*_*i*_ values in the normal liver were 30% lower with the Patlak method than with the 2TC-3k model, while the Patlak distribution volume (*V*_*e*_) values were 30% higher than those based on the 2TC-3k model (*V*_ND_). The kinetics of ^68^Ga-ABY-025 in the liver are not irreversible, which leads to an underestimation of *K*_*i*_ and an overestimation of *V*_*e*_ when using the Patlak method, and an additional reason of the higher Patlak distribution volume is that it includes blood volume, whereas *V*_ND_ does not. Hence, the improved contrast using Patlak *K*_*i*_ is essentially a fortunate benefit of the erroneous assumption of irreversible kinetics in healthy liver tissue, likely resulting in better lesion detection in Patlak *K*_*i*_ images than in SUV images, as clearly shown in Fig. 8. Most peptide-based PET tracers are retained and metabolized in the liver, and as a result, non-specific liver uptake is a common problem in PET imaging. This phenomenon is also observed in recent biodistribution studies [25]. Similarly, preclinical studies investigating the use of other Affibody molecules targeting HER3 receptors also observed non-specific uptake of the given tracer in the liver [26]. Parametric image formation with the enhanced tumour-to-liver ratio is a favourable workaround to this issue not limited to ABY-025 PET but could potentially establish a framework for future studies with similar tracers.

HER2 imaging provides an extended scale to the degree of expression contrary to traditional IHC that incorporates the entire range of positive lesions into the ordinal HER2 3+, as shown in the previous studies [11, 12, 27].

The main limitation of the study is the small number of subjects. Data was used to explore multiple aims, which increases the risk of type 1 errors. There were few lesions with a definite diagnosis from biopsies, and only few patients had HER2-negative disease status. A substantial fraction of lesions were small, which introduces measurement errors. Dynamic studies in the present work were limited to a single bed position, allowing for only 15 cm axial field of view. Multi-bed dynamic PET in current clinical scanners or research total-body PET scanners with automated definition of the input function could allow for performing whole-body parametric imaging and correlative analysis of SUV and T/B ratios towards parametric imaging of the entire body [28, 29]. Future studies should also investigate if the dynamic protocol could be shortened.

## Conclusion

Parametric imaging provided good visualization and eliminated non-specific background uptake in the liver. There was a good correlation of absolute quantification presented by kinetic parameters and the simple SUV from static 2 h and 4 h time point images, further supporting the use of SUV for clinical routine in whole-body imaging and HER2-expression quantification. Besides, *K*_1_ and *K*_*i*_ images provided insight on the pharmacokinetics of ^68^Ga-ABY-025. *K*_*i*_ values might be useful for the quantification of HER2 expression in breast cancer patients under certain conditions, such as anti-HER2 drug development studies and in some clinical cases with known small tumours in the liver. Further studies with a larger patient cohort and more biopsies are warranted.

## Data Availability

The datasets used and/or analysed during the current study are available from the corresponding author on reasonable request.

## Abbreviations

1TC: Single-tissue compartment
2TC-3k: Irreversible two-tissue compartment
2TC-4k: Reversible two-tissue compartment
FISH: Fluorescence in situ hybridization
GMP: Good manufacturing practice
IHC: Immunohistochemistry
*K*_1_: Delivery rate
*K*_*i*_: Net influx rate
OSEM: Ordered Subsets Expectation-Maximization
PET/CT: Positron emission tomography/computed tomography
PMI: Parametric images
SPECT: Single-photon emission computed tomography
SUV: Standardized uptake value
T/N: Tumour-to-normal tissue
TAC: Time-activity curve
*V*_*b*_: Partial blood volume
*V*_*e*_: Distribution volume
*V*_ND_: Non-displaceable volume of distribution
VOI: Volume of interest
